# Train and Reprogram Your Brain: Effects of Physical Exercise at Different Stages of Life on Brain Functions Saved in Epigenetic Modifications

**DOI:** 10.3390/ijms252212043

**Published:** 2024-11-09

**Authors:** Magdalena Kukla-Bartoszek, Katarzyna Głombik

**Affiliations:** Laboratory of Immunoendocrinology, Department of Experimental Neuroendocrinology, Maj Institute of Pharmacology, Polish Academy of Sciences, Smętna 12, 31-343 Kraków, Poland; mkuklaba@if-pan.krakow.pl

**Keywords:** brain, physical exercise, epigenetics

## Abstract

Multiple studies have demonstrated the significant effects of physical exercise on brain plasticity, the enhancement of memory and cognition, and mood improvement. Although the beneficial impact of exercise on brain functions and mental health is well established, the exact mechanisms underlying this phenomenon are currently under thorough investigation. Several hypotheses have emerged suggesting various possible mechanisms, including the effects of hormones, neurotrophins, neurotransmitters, and more recently also other compounds such as lactate or irisin, which are released under the exercise circumstances and act both locally or/and on distant tissues, triggering systemic body reactions. Nevertheless, none of these actually explain the long-lasting effect of exercise, which can persist for years or even be passed on to subsequent generations. It is believed that these long-lasting effects are mediated through epigenetic modifications, influencing the expression of particular genes and the translation and modification of specific proteins. This review explores the impact of regular physical exercise on brain function and brain plasticity and the associated occurrence of epigenetic modifications. It examines how these changes contribute to the prevention and treatment of neuropsychiatric and neurological disorders, as well as their influence on the natural aging process and mental health.

## 1. Introduction

In an era of tremendous technological advancement and the development of specialized bioinformatic tools, significant progress has been made in various fields, including medical research. Among the areas to which special attention has been drawn are epigenetics and epigenome. This growing interest is largely driven by the milestone achievement of completing the first fully sequenced human reference genome in 2022 by the Telomere to Telomere (T2T) consortium [1], which has enabled a more comprehensive understanding of this field [2]. Epigenetics refers to functional and inheritable changes causing modulation of gene expression and phenotypic changes unrelated to DNA sequence modifications [3]. The term epigenetics encompasses DNA methylation, post-translational histone modifications, and non-coding RNA expression [3]. It has been demonstrated that the epigenome remains highly dynamic and environmental factors such as an inappropriate diet, physical inactivity, exposition to pesticides, polycyclic aromatic hydrocarbons, tobacco smoking, radioactivity, bacteria and viruses, and many other adverse factors may act as drivers of changes, which consequently lead to an increased risk of chronic diseases as well as accelerated aging [4]. On the other hand, favorable influences such as nutritional diet, calorie restriction, and physical exercise may positively influence the body systems through epigenetic modifications and significantly improve health [5].

Accumulating data indicate that physical exercise is one of the strongest stimuli that may induce systemic and complex health-promoting effects. However, it should be mentioned that the term “physical exercise”, although sometimes used as a synonym of “physical activity”, actually “is a subset of physical activity that is planned, structured, and repetitive and has as a final or an intermediate objective the improvement or maintenance of physical fitness” [6]. Many studies have demonstrated an association between physical exercise and the reduction of a wide range of disorders across the lifespan, including, i.a., cancers, obesity, and cardiovascular disease [7]. Moreover, exercise was shown to impact the vitality and function of the central nervous system (CNS), having the capacity to reduce cognitive decay in aging as well as exert therapeutic effects in prevention and as a supplementary treatment for neurological disorders [8].

Data suggest that the effects of exercise on cognition may be exerted by affecting molecular pathways associated with energy metabolism, synaptic plasticity, and neurogenesis [8]. Although the exact mechanism of exercise on CNS health is not well established, a growing body of evidence from recent years suggests that beneficial effects of physical exercise are mediated through specific molecules, called “exerkines”, i.e., peptides, cytokines, chemokines, metabolites, and nucleic acids, at least partially released within extracellular vesicles (EVs) from skeletal muscles and other body tissues into the bloodstream, during and shortly after exercise [9]. Due to intravesicular location, exerkines may exert their effects through paracrine and/or autocrine pathways, driving the beneficial outcome on different body organs and providing exercise-induced inter-organ communication [10]. Since EVs may cross the blood–brain barrier, it is also supposed that their cargo from the periphery is able to directly impact the brain tissue [11]. On the other hand, neuron-derived EVs may constitute a good biomarker of CNS condition [12]. The composition of EVs’ cargo released from different tissues varies and may change upon different stimuli. It was shown that both aerobic and resistance exercises may impact the content of these particles, triggering beneficial effects on various body systems [9]. The highly beneficial potential of the EVs’ cargo was demonstrated in some recent studies, in which exercise-induced EVs were injected into sedentary mice, which resulted in a significant reduction in cardiomyocyte apoptosis in vitro and infarct size in vivo [13]. Also, in type 2 diabetes mellitus, exerkines revealed a valuable impact, reducing obesity and improving metabolic health [14]. Importantly, the beneficial effects of post-exercise-released EVs on CNS disorders (depressive disorder) were also reported in numerous research (reviewed in [15]). Although many studies have demonstrated that exerkines encapsulated in EVs efflux from cells constitute a fast response of the body cells to physical training, they do not explain the long-lasting effects of exercise. This in turn may be attributed to epigenetic modifications.

This review discusses recent data regarding epigenetic modifications triggered by physical exercise at various stages of life and collates them with the data on the effects of physical exercise in the prevention and treatment of neuropsychiatric and neurological disorders as well as during continuous processes of natural changes in the CNS.

## 2. Epigenetic Modifications: Mirror of Gene and (Micro)environment Interactions

**Figure 1:**
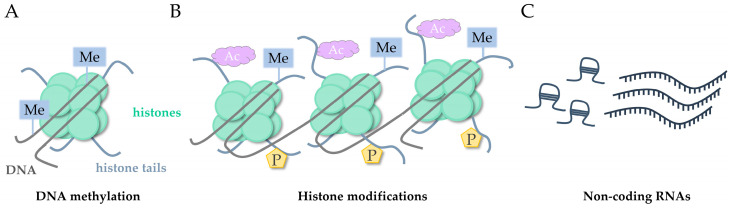
The overview of the main epigenetic modifications impacting gene expression: DNA methylation (**A**); histone modifications (**B**); non-coding RNAs (**C**); Me—methylation, Ac—acetylation, P—phosphorylation.

Data from the last decades prove that the influence of external factors on living organisms is significant. Moreover, it has been demonstrated that some tissues, including the brain, have the ability to keep the effects of environmental stimuli for a long time [16], even for further generations, although this needs further confirmation [17]. Various studies conducted in humans and animal models suggest that epigenetics plays a leading role in this performance. Epigenetic modifications are mechanisms comprising different forms, including DNA methylation, histone modifications, and non-coding RNA expression, all of which are responsible for the modulation of gene expression but also take part in additional processes including DNA damage repair, replication, cell cycle control, degradation, and others. Epigenetics represents a mechanism that explains the phenomenon of different interpretations of the same DNA sequence in different cell types [18]. The overview of the main epigenetic modifications is presented in Figure 1 and is shortly described in the sections below.

### 2.1. DNA Methylation

DNA methylation is a major epigenetic modification, which relies on the transfer of methyl group from S-adenosylmethionine (SAM) to the fifth carbon of a cytosine residue, producing C^5^-methylcytosine (5mC). This reaction is catalyzed by the family of DNA methyltransferases (Dnmts), a class of enzymes considered as “writers”. Dnmt3a and Dnmt3b are responsible for de novo methylation, while Dnmt1 replicates the methylation pattern from the paternal DNA strand onto the daughter strand, which is newly synthesized during the DNA replication process. As a result of the writer’s action, properties of the methylated nucleotides are changed, and stability and affinity for binding partners or methyl-binding proteins (“readers”) were significantly impacted. “Erasers”, in turn, are enzymes responsible for the demethylation process, and together with the previously mentioned writers provide dynamism to the methylation/demethylation process (for further information and review see [19,20]. The majority of methyl group additions occur at sites where cytosine is preceded by the guanine nucleotide in the DNA strand (CpG); however, data are demonstrating a significant portion of non-CpG localization of 5mC [21]. It was estimated that in human somatic cells, 5mC accounts for ~1% of total DNA bases, affecting 70–80% of all CpG dinucleotides in the genome [22]. Apart from modified gene expression, DNA methylation is involved in several other processes observed in mammalian cells, including embryogenesis, retrotransposon suppression, genomic imprinting, and X chromosome inactivation, and plays an essential role in the genome regulation, development, and diseases [23].

### 2.2. Histone Modifications

Histone modification is another mechanism providing a diversity of the outcome when interpreting the information encoded in DNA sequence. It affects chromatin structure, i.e., density, and therefore accessibility of transcription machinery to DNA strand, modifying the transcriptional potential of the underlying DNA sequence. The spectrum of the identified histone modifications is still widening, as well as positions where any modification has been detected, and their impact on biological processes. All DNA-related processes may be regulated by the histone post-transcriptional modifications. The histone proteins are inherently alkaline, forming an octamer composition (H2A, H2B, H3, and H4 highly conserved histones), with 147 base pairs of DNA wrapped around. As mentioned above, this composition may be decorated by the histone modifying enzymes, with post-translational modifications (PTMs), which include methylation, acetylation, phosphorylation, ubiquitination, and others. Due to the reversible character of the reactions of addition and detachment of the specific functional groups to the selected residues predominantly on histone tails, they promote dynamic changes in the chromatin structure, and thus the expression of a specific gene or group of genes, which may appear in response to changing conditions and/or external stimuli. Histone modifications may also impact specific DNA processes, such as DNA repair, replication, or recombination (for more details see reviews [24,25,26]).

#### 2.2.1. Histone Acetylation

The addition of an acetyl group to the lysine residues on histone tails is a reversible modification that is regulated by two classes of enzymes, which are histone acetyl-transferases (HATs, including GNAT, MYST, and p300/CBP) and histone deacetylases (HDACs) [27]. Their activity has been altered in various diseases such as cancers and metabolic disorders. Histone acetylation is considered one of the major regulators of eukaryotic transcription (for review and further information see [28,29]).

#### 2.2.2. Histone Phosphorylation

In contrast to histone acetylation, histone phosphorylation takes place on serines, threonines, and tyrosines, and is controlled by kinases and phosphatases, which add and remove phosphate groups, respectively. Most of the phosphate group additions are identified within the N-terminal tails of the histones, though not exclusively. Histone phosphorylation has been predominantly associated with cellular response to DNA damage; however, crucial roles in chromatin remodeling linked to other nuclear processes have also been reported by multiple studies [30,31].

#### 2.2.3. Histone Methylation

This modification is predominantly observed on the side chains of lysines and arginines. On the contrary to previously described modifications, histone methylation does not change the charge of the histone protein. Moreover, to further increase the level of complexity, single positions may be mono-, di-, or tri-methylated. Depending on the amino acid that undergoes modification, histone lysine and arginine methyltransferases and demethylases are involved in these reactions [32]. It was demonstrated that histone methylation contributes to biological processes associated with development and cellular response [33].

#### 2.2.4. Other Histone Modifications

Less common but with similar potency are also other histone modifications including ubiquitination, sumoylation, ADP ribosylation, deimination, and proline isomerization. Analogical to previously described, these modifications also contribute to DNA-related processes, such as DNA repair, replication, recombination, and others [34,35,36].

### 2.3. Non-Coding RNAs

Non-coding RNAs (ncRNAs) as a type of epigenetic regulation have been observed more recently and refer to clusters of RNAs (including short: miRNA, piRNA, siRNA, and long non-coding RNA) that are not involved in functional protein encoding [37]. Recently, novel groups of ncRNAs have been described: promoter-associated (PARs) and enhancer RNAs (eRNAs) [38]. NcRNAs play two main roles—functional and regulatory. They participate in gene expression regulation, impacting numerous biological processes including cell growth and differentiation through various ways, i.e., modifying chromatin function or interfering in the stability and translation of transcripts. Non-coding RNAs constitute a promising target of therapy as well as they may serve as biomarkers since their expression is often characteristic of particular tissues and conditions [39].

## 3. Physical Exercise at Different Stages of Life Impacts Mental Health and Brain Functions via Epigenome Modifications in the Brain

An increasing amount of evidence demonstrates that physical exercise can serve as a powerful intervention to enhance mental well-being and cognition, promoting changes at the molecular level in the brain (for review see [16]). Indeed, these alterations contribute to improved synaptic plasticity, the neurogenesis process, cognitive functions, and mental health, illustrating the profound impact of lifestyle factors on brain biology. The study is ongoing and the molecular mechanisms behind these changes/adaptations are yet to be fully elucidated. Up to date, data have shown that modifications collectively called epigenetic alterations may be a driver of these adaptations, as they were recognized in various tissues in response to physical exercise [40,41,42]. Orchestrated activation of various mechanisms and signaling events leading to the transcriptional and translational regulation, at least in part, which are the result of high flexibility of the epigenome, constitutes the body’s integrated response to exercise.

The most intensively recognized epigenetic modification induced by physical exercise is DNA methylation. Studies showed that the effect of exercise highly depends on its character, intensity, duration, and tissue/cell type. Moreover, it was shown that regular exercise promotes hypomethylation of CpG sites in the *Bdnf* promoter, resulting in a stable increase in *Bdnf* expression, which in turn improves hippocampal synaptic plasticity, learning, and memory [43]. Various studies proved that other epigenetic changes such as histone modifications, and non-coding RNA expression may also be relevant. The previously mentioned study reported by Gomez-Pinilla, et al. [43] demonstrated that acetylation of histone H3 in the *Bdnf* promoter IV, along with a reduction of Hdac5 levels, resulted in the transcription and stable *Bdnf* expression in the rats’ hippocampus. Another study revealed altered activity of Hats and Hdacs and increased Hat/Hdac balance for both H3 and H4 histones one hour after treadmill exercise in the hippocampus of Wistar rats [44]. Based on these and other results, the authors suggest that the neuroprotective effects of physical exercise may be associated, at least in part, with the hyperacetylation status of histones, which leads to high transcriptional activity [44]. Also, non-coding RNA expression modulation in the brain has been reported in response to physical exercise. Especially, the level of some particular miRNAs has been reported to be changed upon physical exercise [45]. Recently, circulating miRNAs have gained much attention due to their potential for mediating cell–cell and tissue–tissue cross-talk (e.g., [46]). Very recent data reported also, that miRNAs encapsulated in extracellular vesicles may be released upon physical training from various cells and tissues into the bloodstream, and, due to the ability to cross the blood–brain barrier, get to the brain (for review see [15]).

**Figure 2:**
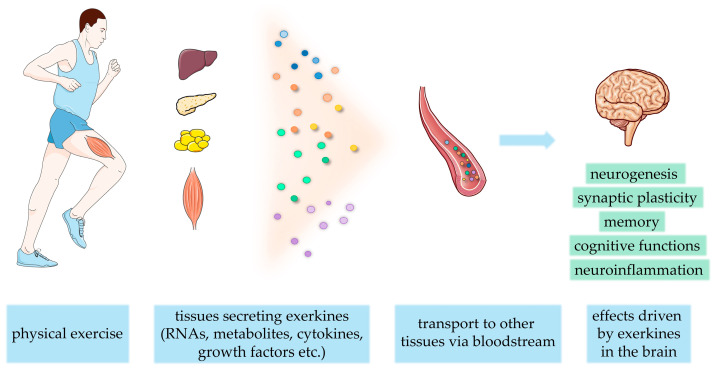
Physical exercise triggers a release of exerkines into the bloodstream from different body tissues during and right after the activity, such as RNAs, metabolites, cytokines, and growth factors. They can cross the blood–brain barrier and exert direct effects on the brain. The figure was generated using Servier Medical Art (Servier; https://smart.servier.com/; access date: 8 October 2024), licensed under a Creative Commons Attribution 4.0 Unported License.

The relationship between physical exercise and brain functions has been extensively studied, however, research has predominantly focused on aerobic exercise to demonstrate that physical activity significantly contributes to neurogenesis and cognitive function (for review see [47]). Nevertheless, each of the three main types of exercise i.e., aerobic, anaerobic, and resistance, has been shown to be potent stimulators of brain plasticity and cognitive function, though the specific mechanisms driving these changes may vary across the different exercise modalities [48]. The benefits of aerobic and anaerobic exercises were associated with changes in the levels of e.g., BDNF, lactate, and vascular endothelial growth factor (VEGF). On the other hand, resistance exercise appears to enhance brain plasticity through the action of myokines such as irisin, insulin-like growth factor-1 (IGF1), and BDNF, which are secreted from skeletal muscles and promote neurogenesis in the brain. Additionally, the intensity and duration of the training, and rest intervals provide distinct stimuli for the brain [48]. The influence of physical activity on the body’s systemic response and exerkines release is presented schematically in Figure 2.

For obvious reasons, most of the studies on the epigenetic changes in the brain triggered by physical exercise come from studies on animal models. Since this review addresses the effects of exercise at different stages of life, in the next sections, their effect on the brain, concerning the time when they were performed, is described. We tried to provide insight into the observed molecular changes in the offspring brain with particular emphasis on epigenetic changes (Table 1).

### 3.1. Preconception Period

Accumulating evidence demonstrates that preconception lifestyle and environmental stimuli impacting one or both parents can significantly modify the physiological and behavioral phenotypes of their progeny and that the good health status of parents is a biomarker of the health of their children. Although the consequences of inappropriate behaviors and health habits during the preconception period can extend across generations, awareness of the existence of such associations is not commonly known [74]. A series of interventions known as preconception care were designed to recognize and address biological, behavioral, and social concerns in women who are or will become pregnant. By managing risk factors that impact pregnancy outcomes and therefore the health of future generations, as well as preventing disease, preconception care aims to enhance the health of women during their reproductive age and the outcomes of their pregnancies [75]. For instance, studies showed that birth weight may be associated with maternal blood pressure, obesity, depression, or substance use, while gestational age depends on maternal body mass index, diabetes, and blood pressure [76]. Although less is known in this context, also paternal health conditions including, i.a., obesity or cardiovascular health are highly important and, most probably via epigenetic mechanisms, impact the birth outcome and the offspring’s long-term health [76]. As the health status of future parents is largely a consequence of lifestyle habits and behaviors, as well as various interventions undertaken at that time, special attention to this topic should be paid to improve the health of mothers and children and minimize the rising incidence of non-communicable disorders [74].

Effects of various conditions such as undernutrition, BMI, or factors such as smoking, alcohol, caffeine, micronutrient supplementations, and many others, during preconception time, have already been investigated (for systematic review see e.g., [77]), but preconception care practices also include physical activity. The beneficial effects of physical exercise of fathers and mothers during the preconception period on the mental health, learning, memory, and cognitive ability of the offspring were recognized. It was demonstrated in the C57BL/6 mice that paternal treadmill exercise (60 min/day of treadmill training, 5 days/week) for 6 weeks before mating improved spatial learning and memory, and induced overexpression of reelin and Bdnf in the hippocampi of male pups [78]. In line with this, McGreevy and colleagues reported that moderate exercise (treadmill training of C57/BL6J male mice; 12 m/min for 40 min/day, 5 days/week for 6 weeks) induced paternal cognition enhancement that was further ‘inherited’ by the offspring, along with the elevated adult neurogenesis, increased mitochondrial citrate synthase activity, suggesting specific reprogramming of hippocampal mitochondria, and a modified profile of adult hippocampal gene expression [79]. The study pointed out that paternal physical exercise may constitute an important factor affecting the offspring’s brain physiology and cognition later in life. Although no exercise-induced changes in DNA methylation of father sperm cells were identified, the GSEA analyses suggested possible mechanisms of epigenetic inheritance associated with the action of specific miRNAs [79].

Supposing that epigenetic modifications determine a highly possible mediator of the inherited beneficial effects of physical activity, a series of studies investigated the impact of physical exercise on the level of hippocampal DNA methylation. In one such study involving adult male Wistar rats, paternal exercise including 20 min/day of running on a treadmill for 5 consecutive days/week for 8 weeks prior to mating was implemented and it was found that the training triggered a decrease in global hippocampal DNA methylation in the runners’ offspring, compared to offspring of sedentary fathers [49]. Further studies of this group [50] applied similar training rigor and animal model (Wistar rats; 20 min/day of running on a treadmill, 5 consecutive days/week for 22 days) and confirmed a significantly decreased level of global hippocampal DNA methylation in the progeny of exercised fathers, which was accompanied by an improvement in spatial learning. Nevertheless, no changes in neuroplasticity biomarkers in the hippocampus (Bdnf, reelin, and BrdU) were observed. This study confirmed a link between paternal preconception sport practice and offspring’s cognitive benefit, and possible involvement of hippocampal epigenetic programming in male offspring, although the exact biological mechanism of this regulation remained not fully understood [50].

The mother’s lifestyle and behavior during preconception time cannot be neglected either, and apparently, similar mechanisms may be involved in the future offspring’s brain programming. One of the studies investigating the impact of exercise on offspring’s brain functions and molecular factors expression involved treadmill running (20 min/day for 5 consecutive days/week for 22 training days; 30% VO_2_ max) of Wistar female rats during the pregestational period. Besides the improved plasticity and spatial cognitive ability, the offspring of mothers subjected to physical training revealed also a decrease in global hippocampal DNA methylation as well as a lack of differences in the Bdnf level when training and sedentary groups were compared [51], showing similar effects to these observed previously in the case of the offspring of exercising fathers. These studies clearly showed that the impact of both parents is evident; however, it remains to be elucidated whether the mother’s and father’s effects operate independently or jointly, impacting the final health status of the offspring [80].

Over time, the question arises as to whether resistance preconception exercise can also produce effects similar to those of aerobic training. Studies on rodents demonstrated an increased expression of offspring hippocampal neuroplastic markers (BrdU+ and IGF-1), as well as a histone H4 acetylation as a result of such training (physical resistance exercise protocol consisted of climbing voluntarily a vertical ladder). In agreement with previous results, a decrease in global hippocampal DNA methylation in the offspring of training dams was also reported [52]. Interestingly, offspring of dams who continued training during pregnancy did not reveal additional modifications in plasticity or epigenetic parameters. Overall, it was demonstrated that not only aerobic but also resistance exercise performed by mothers during preconception time may have a beneficial effect on offspring brain functioning [52].

Importantly, preconception physical exercise may not only provide brain health for the offspring, but it can also act preventively in the context of the consequences of future negative and/or stressful events. Studies on rodents showed that regular physical exercise before the gestational period may attenuate the detrimental effects of stress experienced during the pregnancy and its adverse impact on fetal brain development via epigenetic mechanisms. In particular, prenatal stress altered histone H3 acetylation, leading to its decrease, while exercise before gestation inhibited this effect. However, the study’s authors suggest that some of such effects may be sex-dependent, as only male offspring revealed a decrease in histone H3 acetylation in the prefrontal cortex of male mice [53]. It was shown that exercise can reduce HDAC activity in the prefrontal cortex and enhance histone H3 acetylation in the hippocampus and cerebellum [57,81]. Since the balance of HDAC and HAT is critical for neuronal homeostasis and, consequently, appropriate brain function [82], modifying HDAC activity and H3 acetylation would provide insight into the mechanism of the impacts of pregestational exercise on effects on stress during pregnancy. The gathered data demonstrate that studies examining the impact of physical exercise during preconception time on gestational adverse stimuli should be continued to precisely determine physical training importance and the necessary frequency and duration to achieve expected beneficial effects on the offspring.

### 3.2. Gestational Period

The gestational period is the other highly important time when environmental factors and the mother’s body condition may significantly impact the offspring’s health. The World Health Organization (WHO) published recommendations regarding antenatal care, showing strong evidence that either diet or exercise, or both, can lower the risk of high gestational weight gain during pregnancy [83]. A decreased risk of macrosomia, cesarean birth, and infant respiratory morbidity are possible additional advantages, especially for high-risk women undergoing combination diet and activity therapy. Exercise seems to play a significant role in managing weight gain during pregnancy, but additional studies are required to determine acceptable standards and indicate the mechanisms underlying this phenomenon (for review see [84]).

Not only weight gain but also neurobiological consequences of maternal training were previously explored and determined. Indeed, numerous data from rodents showed the beneficial effects of physical exercise of mothers during pregnancy on brain functions, cognition, and mental health of offspring. A study, conducted on Sprague-Dawley rats subjected to running on a treadmill (30 min/day, 5 days/week, 20 m/min), revealed increased levels of hippocampal *Bdnf* transcripts on postnatal day (PND) 0, co-observed with significantly improved spatial learning ability in pups on PND 47 [85]. Further examination involving pregnant rats who were forced to swim 10 min/day revealed, again, increased hippocampal neurogenesis and *Bdnf* expression in the offspring, as well as improved short-term memory capability [86]. Also, the same research group disclosed that maternal exercise, which involved mild running on a treadmill, 30 min/day beginning on the 15th day of pregnancy until delivery, resulted in increased *Bdnf* transcript levels, enhanced hippocampal cell survival, and improved short-term memory of rat pups (PND 29) when compared to the control group [87]. In fact, the modulatory effect of *Bdnf* on brain functions, such as memory and cognition, resulting from physical activity, has been well recognized, and interestingly, the attenuation of its expression was found to be related to depressive-like behavior [88]. Also, the effect of training on learning and memory processes was well recognized by various studies. For instance, one such involved both: voluntary running on a running wheel and swimming (10 min/day until the end of the pregnancy) of pregnant rats. Besides facilitated learning, an increased number of cells in the cornus ammonis 1 (CA1) and dentate gyrus (DG) hippocampal regions were identified [89]. This increased quantity of neurons in the hippocampal subregions as a result of maternal running exercise was further demonstrated by other studies. Gomes da Silva and colleagues showed an increase in the number of neurons in the hippocampal formation, but not cerebral cortex [90], and Dayi and co-authors in the hippocampus of prepubertal and adult rats [91]. The latter study also revealed increased hippocampal cell survival and improved learning memory capability in these animals [91]. Research on the impact of physical activity also concerned other brain structures, including the prefrontal cortex, a brain structure that plays a crucial role in emotion regulation. Regular mild treadmill exercise throughout pregnancy significantly increased levels of Bdnf but also Vegf on PND 26- and in 4-month-old rats, both male and female [92]. Moreover, the positive correlations between Bdnf levels and open field tests as well as Vegf levels and elevated plus maze test were demonstrated, suggesting that prenatal physical exercise may, through regulation of these factors, protect against anxiety in offspring [92]. Although all these studies demonstrate that prenatal physical activity may significantly impact offspring brain formation and functions, mostly through the modulation of the Bdnf level, they do not provide unequivocal evidence as to what the biological mechanism behind these intergenerational benefits of physical activity might be hidden.

Further research took a step forward in this context, showing an association between better performance in learning and memory tasks directly with epigenetic modifications. Lower Hdac2 amounts in the offspring of mothers subjected to resistant exercise (climbing voluntarily a vertical ladder, with lead weights fixed to animal tail) during pregnancy, have been linked with improved memory and learning skills [52]. These results stay in line with other reports providing evidence that Hdac2 negatively regulates brain functions such as memory formation and synaptic plasticity, while Hdac2-specific inhibitors constitute potential treatment strategies for those with memory-impairment-related diseases [93]. Moreover, as a regulator of many neuronal gene expressions, as well as one of the key regulators of neuronal development, HDAC2 level alterations were implicated in various abnormalities including neurodegeneration, but also brain aging [94,95,96]. In another study, physical exercises of Wistar pregnant dams running on a treadmill (5 consecutive days/week for a total period of 22 training days; 20 min/day 60% VO_2_ max) triggered improved spatial learning and plasticity in offspring that was manifested by the increased number of BrdU+ and reelin+ hippocampal cells. However, the changes in the level of DNA methylation were not observed as a result of gestational training [51]. Therefore, it seems that the type of training is essential in the context of modulating epigenetic changes induced during pregnancy.

Physical exercise during pregnancy was also evaluated in the context of its preventive action against the effects of prenatal adverse stimuli and conditions. The problem of advanced maternal age has been previously addressed in some animal studies, which showed that the advanced age of the mother during delivery was significantly associated with impaired learning, memory, and hippocampal Bdnf levels as well as reduced expression of markers related to cell proliferation, survival and migration in the hippocampus [97,98,99]. Therefore, the potential preventive effects of physical exercise during pregnancy and the positive effect on the cognitive function of offspring were assessed. The 10-month-old C57BL/6 female mice (along with younger counterparts) were subjected to treadmill running (in the following rigor: 3 m/min for 5 min, 5 m/min for the next 5 min, and 8 m/min for the last 20 min, 0° inclination; 6 days/week, for 8 consecutive weeks before mating and during pregnancy) and it was found that maternal training improved hippocampal neuroplasticity, spatial learning, memory, and mitochondrial functioning as well as increased hippocampal levels of Bdnf, preventing negative consequences of advanced maternal age on the cognitive functions in their offspring; however, the mechanisms of such a therapeutic effect was not resolved [99]. In another similar study, the impact of physical exercise involving running on a treadmill (60 min/day, 5 days/week, 3 weeks) on the adverse effect of sevoflurane, a volatile anesthetic used during surgical interventions, was evaluated in Sprague-Dawley rats. As a result, cognitive dysfunctions in the offspring induced by the neurotoxic effects of sevoflurane administration during a gestational period were alleviated.

Moreover, reduction in p300 Hat expression as well as levels of acetylated histones (ace-H3K14, ace-H3K27) in the hippocampi of offspring rats was reported to be ameliorated by prenatal training, suggesting highly possible epigenetic mechanisms involved in the exercise-induced prevention [54]. p300, a transcriptional co-activator, has been linked to synaptic plasticity and memory processes via its Hat activity, whereas excercise-stimulated p300 Hat expression and histone acetylation modifications at H3K9 and H3K27 in offspring may be linked to their neuroprotective effects. Moreover, the authors established a relationship between p300 HAT-mediated histone acetylation and Bdnf/TrkB signaling in maternal exercise-induced neuroprotection. As already mentioned, Hat/Hdac imbalance has a significant effect on the development of various brain disorders, which gives further arguments for the beneficial impact of physical training in the prevention and treatment of neuropsychiatric disorders.

Although the substantial impact of factors acting during gestation on the fetus is obvious, it seems that factors affecting the newborns just after birth are of no less importance. Recent studies widely describe the role of early life nutrition, with the central dispute on breastfeeding, which presumably protects against the development of obesity and other metabolic diseases later in life, when the mother’s diet is balanced [100]. As data showed, maternal diet and probably lifestyle may significantly influence breast milk composition (e.g., [101,102]). Although there are few data in this area, recent investigations suggest that maternal exercise also may help protect against the onset of various diseases, impacting breast milk composition, that may mediate metabolic and cardiovascular health in the offspring [103]. Although some breast milk components are recognized to boost newborn brain development [104], the issue of the potential transfer of exercise-induced compounds/RNAs through the milk from mother to offspring and its effects on their brain health poses questions for researchers and has yet to be determined.

### 3.3. Early Years

The early period of life is especially important for brain health. It is a stage of accelerated physical and cognitive development, involving the rapid proliferation of neurons and the formation of new neural connections and synapses. Therefore, appropriate care for the CNS should be provided at this stage. Engaging kids in physical activity is highly effective, and with almost no risk or side effects powerful intervention, that can have a long-lasting impact on their brains, improving lifetime brain functioning and reducing the chance of brain disorders in the future. According to WHO recommendations, children and adolescents should spend “at least 180 min in a variety of types of physical activities at any intensity, of which at least 60 min is moderate to vigorous intensity physical activity, spread throughout the day”. Numerous studies reported beneficial effects and positive correlations between physical activity during childhood and preadolescence and academic achievements and/or cognitive functions [105]. However, a consensus about the role and exact portion of physical activity influencing academic-related outcomes in early childhood remains elusive, largely due to the wide variation in methodological approaches [106,107]. Also, a very early life exercise impact on brain epigenetic landscape has not been well investigated so far, and undoubtedly this needs further investigation; however, this effect has been studied more in adolescents.

Adolescence, a critical period of neural maturation and neurodevelopment, is linked with massive reorganization of the CNS and accompanied by improved cognitive function. Epigenetic processes are highly important in further CNS development and neural cell differentiation, as well as the integration of social, hormonal, environmental, and genetic information during this phase of life [108]. It was found that a neotenic shift in gene expression during brain maturation in humans leads to increased epigenetic regulation, extending neural plasticity and providing additional time to learn and develop abilities [109]. There are also significant differences in the developmental epigenetic programming between neurons and glia. In particular, during periods characterized by rapid synaptogenesis, such as in adolescence, the epigenome undergoes significant reconfiguration [110]. Rodent studies indicate that specific subgroups of microRNAs in the brain are expressed differently during developmental stages, increasing during fetal growth, decreasing as the brain matures, and decreasing in adulthood. Probably, it is necessary to diminish particular protein levels at critical time points to facilitate important developmental processes [111]. The sex- and region-dependent gene expression in the adolescent brain was also described. For instance, estradiol exposure epigenetically modulates expression of its receptors in the preoptic area of the brain (crucial for sexual and parenting behaviors) and in consequence, females display 30% higher levels of methylation of these factors than males, whereas microRNA-mediated gene expression changes in response to gonadal hormones lead to prenatal brain masculinization [112].

Moreover, adverse prenatal events such as stress, diet, drug exposure, and bacterial/viral infection can cause significant epigenetic changes in the adolescent brain. It is known that prenatal stress, early life stress, and addiction have a significant impact on teenage brain maturation and the epigenome. What is more, sedentary behavior during adolescence, often caused by excessive use of electronic devices, and social or audiovisual media increases risk not only for overweight or obesity but also may be linked with reduced intelligence [113]. Young people aged 10 to 24 years represent 24% of the world’s population, and investigating their health has revealed that the majority of them do not perform enough physical activity. The problem is spreading worldwide and can have major consequences for health and well-being, so immediate action is required to reverse this trend.

Rodent studies demonstrated that just after one week of training (voluntary wheel running), global acetylation of histone H3 increased in the hippocampus and cerebellum of young mice and this observation in the hippocampus correlated with increased *Bdnf* level. At the same time, the expression of *Dnmts* and *Hdacs*, genes that modify DNA methylation and histones, was diminished in both brain areas after training [57]. Enhanced levels in histone H3 phospho-acetylation in hippocampal DG area of young Sprague-Dawley rats in response to voluntary exercise (4 weeks of training on a running wheel) were observed also by Collins and co-authors [55]. In addition, this change was linked with increased stress-coping capabilities and memory performance seen in exercised animals. Such data are highly significant, but there are only a few studies that focus on the impact of exercises on postnatal hippocampal maturation (particularly, synaptic rearrangement and plasticity). The study of Ivy and co-authors [114] conducted in a mouse model of early-life voluntary running on a wheel demonstrated that exercise from the fourth to sixth postnatal weeks (juvenile-adolescence), and one month during the juvenile period, facilitates hippocampal-dependent spatial memory development, synaptic plasticity, and alter hippocampal excitability in mice, but it is not known whether observed changes are linked to the epigenetic modifications. However, a subsequent study, conducted recently by this group, showed that histone modification may be essential in memory facilitation after early life exercise (ELE) [58]. It was demonstrated that ELE regulated H4K8ac and H3K27me3 occupancy at regulatory regions of genes involved in hippocampal memory consolidation. Similarly, histone H4K8 acetylation at *Bdnf* promoters I and IV in 6-week-old male mice (C57Bl/6J) was increased in response to 3 weeks of exercise with the use of a running wheel [56]. Despite limited studies, it appears that exercise in childhood and even more during adolescence is an efficient intervention with the potential for generating beneficial, long-lasting gene expression modifications connected with synaptic plasticity, signaling pathways involved in learning, enhanced memory, and cognition.

### 3.4. Adulthood

Methylome undergoes significant rearrangement throughout change from the fetal phase through childhood to adulthood, which is closely linked to the synaptogenesis process. During this age-related transition, highly conserved non-CG methylation (mCH) becomes accumulated in neurons, but not glia, to transform into the dominant form of methylation in the human neuronal genome [110]. The change of the neuronal DNA methylome was for a long time an underappreciated mechanism for activity-dependent epigenetic control in the adult nervous system, but studies have shown that epigenetic DNA modifications may increase the ability of mature neurons to convert temporary signals into long-lasting modifications in the nucleus. Considering the epigenetic disturbances associated with aging, neurological, and mental illnesses, the reversibility of DNA methylation—triggered by various stimuli in the adult brain through neuronal activation and behavioral stimulation—presents a promising avenue for developing new treatments [115].

Studies demonstrate that various environmental and genetic factors (including micronutrients, air pollutants, harmful substances, specific habits, and behavior) may exert a significant impact on brain functions via epigenetic machinery also during adulthood, sometimes with long-lasting effects (for review see, e.g., [116]). In connection with this, numerous studies indicated strong associations between adult brain health, epigenetic mechanisms, and physical exercise. Exercise protocol including 7 days of voluntary wheel running of adult Sprague-Dawley rats induced DNA demethylation at *Bdnf* promoter IV, a region highly responsive to neuronal activity, and consequently increased *Bdnf* levels in the hippocampi of tested animals [43]. This was accompanied by the increased MeCP2 protein level and histone H3 acetylation, and a reduction of Hdac5 transcript and protein levels in the hippocampus [43]. Although, as mentioned in previous sections, substantial evidence suggests that the elevation of Bdnf levels is a key factor through which exercise enhances synaptic function, multiple pathways and mechanisms, including epigenetic regulation, likely contribute to this effect. However, some studies provided insight into this process, and one of them subjected mice to 30 days of voluntary running. It was observed that physical activity elevated Bdnf levels in mice hippocampi and induced the production of B-hydroxybutyrate in the liver, which was subsequently transported to the brain, leading to reduced levels of *Hdac2* and *Hdac3* genes in the hippocampus [60]. Indeed, a growing body of evidence indicates that physical exercise induces the production and release to the circulation of a wide variety of signaling, humoral factors, similar to B-hydroxybutyrate, called “exerkines” (or “myokines” when released by the muscles), from different body tissues, which then may act in auto- and/or paracrine way, triggering specific, including epigenetic, changes in the recipient cells [9]. Epigenetic modifications being a result of exercise were described in various tissues, especially in muscles, but also adipose tissue, liver, pancreas, and brain, where they constitute a part of complex adaptation aimed at improvement of the performance of the body [41]. This exercise-induced interorgan crosstalk, as it is currently considered, was recently widely described [117,118].

The mechanism underlying physical exercise-induced beneficial effects on brain functions seems to be even more complex. The involvement of the acetylation process in the beneficial effect of exercise on the brain is widely highlighted. In one such study, mice were subjected to isoflurane neonatal exposures, which resulted in the deregulation of histone H4K12 acetylation [64] but the protective role of swimming exercise in this process was observed. The training, consisting of swimming for 4 weeks, four sessions of 5 min/day, 3 months after anesthesia, resulted in enhanced acetylation of selected positions, including H3K9, H3K14, H4K5, H4K8, and H4K12. Moreover, H3K9 was stable for up to 4 weeks [64]. Studies also showed that voluntary exercise exposure (wheel running (P56-P95)), following adolescent intermittent ethanol, restored the loss of cholinergic neuron markers (ChAT, TrkA, and p75NTR) in the adult basal forebrain and this effect was mediated by histone (H3K9) modification and DNA methylation mechanisms, confirming that epigenetic changes may underlie learning improvement and cognitive abilities [66]. These studies again demonstrated the protective role of exercise, which is often associated with changes based on epigenetic modifications, including histone acetylation, and can be implemented successfully even in adulthood.

Data insights into the regulatory mechanisms of epigenetic mechanisms in response to physical training as behavioral stimuli revealed that exercise can also change the miRNA expression profile in the brain. A 6-week swimming training applied to rats (a high-intensity intermittent training: 6 weeks, 10 sessions of swimming per day, 6 min each) resulted in upregulation of all miR-200 family members, which play a regulatory role and exerted neuroprotective, and pro-neurogenesis effects in the brain (especially in examined areas: cerebrum and cerebellum) [65]. The study showed that many exercise-responsive genes (i.e., *Bdnf*, *Igf*-*1*, *Ggf*, *c*-*Fos*, *Ntf3*, *Ntf4*, and *Vgf*) may also be targeted by exercise-responsive miRNAs. Also, mice subjected to traumatic brain injury (TBI) were characterized by altered miRNA expression in the hippocampus. Moreover, it was revealed that miR-21 or miR-34a are associated with the recovery process induced by running training (voluntary running wheel), and therefore it was proved that an epigenetic mechanism might be involved in exercise-induced cognitive enhancement of mice that suffered from TBI [45].

As already described in the previous section, physical activity during adulthood has also been identified as an effective intervention for the prevention of a range of disorders [119]. Various biochemical pathways and epigenetic mechanisms have been proposed to explain the positive impact of physical exercise on cognitive function and health [120]. Moreover, regular physical activity in adulthood may significantly impact brain functions and health during aging. A study performed in adult and aged C57BL/6 mice demonstrated that 8-week physical activity including voluntary running (running wheels) induced anxiolytic and antidepressant effects [121], probably due to hypomethylation of the RE1-silencing transcription factor (*Rest*) promoter in the hippocampus [120]. Increased *bdnf* and *Rest* expression along with decreased IL-1b and IL-10 were also observed in aged mice as a result of running as an adult. Indeed, physical exercise, as one of the most effective, non-pharmacological interventions to maintain a healthy brain, has been used as a treatment for 26 chronic diseases, including depression, anxiety, and schizophrenia [122]. The possible mechanism of the therapeutic effect of exercise in major depressive disorder, involving exerkines and miRNAs transported in extracellular vesicles, was proposed [15]. Moreover, exerkines, including some miRNAs, were also found to be involved in the alleviating effects of early life stress, encompassing cognitive impairment and depression-like behavior [123]. On the other hand, decreased *Dnmt1* expression and increased global DNA methylation were reported in the brains of animals submitted to swimming during 53–78 PND followed by repeated restraint stress (RRS) during 75–79 PND. Additionally, research on the social defeat-induced stress model has demonstrated that exercise, such as 30 min a day of treadmill running for two weeks, restored the alterations in the hippocampus caused by stress on histone H3 acetylation, HDAC5, and MeCP2 [61]. It was also reported that 4 weeks of physical-exercise induced (voluntary exercise, running wheel) the up-regulation of *Bdnf* transcripts in mice subjected to further RRS, which was accounted for by the increase in histone H3 acetylation at specific *Bdnf* promoters [62]. These studies demonstrated that exercise may modulate epigenetic responses in the brain triggered by the stress experienced in adulthood. Taking all this together, physical exercise may constitute an effective therapeutic and preventive strategy in a variety of neuropsychiatric disorders and its significant impact on epigenetic regulation in the adult brain should be taken into account.

### 3.5. Aging and Neurodegeneration

Aging is characterized by progressive functional decline at the molecular, cellular, tissue, and organismal levels. As a consequence, mental and physical performance continues to decrease, increasing the risk of illness and finally death. However, the average lifespan has significantly increased mostly as a result of great scientific developments in biology and medicine during the past century. The unprecedented growth of the older population has created a demand for the development of innovative treatments for the prevention, early identification, and treatment of aging-related disorders, and disabilities. Since aging is regarded as the final stage of development, deregulation of epigenetic controls is hypothesized to play a role in age-related diseases, such as cognition impairment [124], cardiovascular disease [125], type 2 diabetes [126], and neurodegenerative disorders [127]. Epigenetic changes have been demonstrated to have a major impact on aging, contradicting the widely held belief that mutations are the major cause of this process. However, it remains probable that these epigenetic modifications are a result of the condition rather than its primary cause. Furthermore, both the aging and the epigenetic changes may be influenced by common environmental or genetic factors (reviewed in [128]. During aging, the genome undergoes global and local DNA methylation alterations. Global DNA hypomethylation is a common manifestation of aging, although specific CpG islands can also exhibit hypermethylation. What is more, numerous age estimators = “epigenetic clocks” (i.e., the single-cell age clock (scAge), PhenoAge, GrimAge, Horvath’s clock, and Hannum’s clock) were developed based on changes in DNA methylation within the genome. By using a set of CpG sites, whose DNA methylation statuses are constant throughout a variety of cells, tissues, and organs, these clocks are able to estimate an individual’s age and predict their chance of developing age-related disorders [129]. In addition, all aging models exhibit overall histone loss and global chromatin rearrangement. The most explored of these alterations and ones that are known to be associated with aging are methylation and acetylation at lysine residues. Global aging-related alterations in H3K9me3, H4K20me3, H3K27me3, and H3K9ac levels have been established in both in vitro and in vivo studies [130]. Because of the altered chromatin accessibility, it results in the reactivation of repetitive sequences and dysregulated gene expression. During cellular senescence, significant chromatin structural remodeling has been found, ranging from modifications to histone components and modifications to changes in chromatin compartments and topologically associating domains (TADs). However, global canonical histone loss has been reported to be a prevalent characteristic of aging (reviewed in [131]). Moreover, RNA modification (mainly in m6A modification, m5C modification, and adenosine-to-inosine (A-to-I) editing) and ncRNA (microRNAs (miRNAs), long non-coding RNAs (lncRNAs), R-loops, and circular RNAs (circRNAs)) regulation also play important roles in cellular senescence via post-transcriptional mechanisms ([132,133] and references therein). The study of RNAs’ regulatory functions in aging now is a rapidly expanding field because for a long time the vast majority of studies were mainly focused on protein-coding genes. A very interesting hypothesis was proposed by Brewer and coworkers [134]—the epigenetic oxidative redox shift (EORS) theory of aging. According to EORS, a lack of activity associated with aging results in a diminished mitochondrial function and an oxidized redox shift. To maintain resting energy levels, redox-sensitive transcription factors increase aerobic glycolysis.

Since epigenetic alterations can generally be reversed with the application of epigenetic regulators, these modifications offer also an attractive target for aging intervention techniques. One such intervention is physical activity/exercise, recommended to the older population since it prevents or improves managing the majority of the most common challenges that older persons deal with, including pain, diminished mobility, frailty, cognitive decline, and many more. Although daily aerobic, strength, balance, and flexibility components are part of a well-balanced exercise program that combats aging, most elderly people do not meet the suggested minimum of minutes of regular physical activity each week (reviewed in [135]).

Studies on aged mice showed that aging-associated decreased hippocampal levels of translocation methylcytosine dioxygenases (*Tet1* and *Tet2*) implicated in the active demethylation of DNA can be counteracted by voluntary exercise (running wheel for 4 weeks), and this change may be involved in the memory improvement showed in behavioral tests [71]. However, it seems that running on a treadmill (single session and/or chronic protocol) did not modify the generation of genomic methylation patterns by Dnmt1 nor Dnmt3b levels in 20-month-old rats’ hippocampi, suggesting that exercise might not influence the transcription activity through Dnmt activity in aging brain. On the other hand, it was observed that the single exercise session reversed the diminished H3K9 methylation levels in aged animals, and it is therefore supposed that running can positively alter the transcriptional activity through mono-methylation of H3-K9 [59]. However, Ionescu-Tucker and colleagues’ results [73] showed that exercise reduced H3K9me3 at *Bdnf* promoter VI in aged mice, but did not significantly change H3K9me3 levels at the promoters of neuronal plasticity genes, which may indicate that H3K9me3 promoter repression is not the main factor influencing neuronal plasticity gene expression (at least in aged brains).

Moreover, physical exercise alters the epigenetic modifications in the course of neurodegenerative processes that are associated with aging. Based on the studies, exercise has been proposed as an epigenetic protector against brain aging and neurodegeneration because it has been demonstrated to exhibit beneficial anti-aging properties through improving neurogenesis, neuro-vascularization, and neurotrophic factor synthesis [136]. It has been shown that running promotes neurogenesis, which facilitates the recovery of synaptic plasticity as well as memory capacity impaired in the process of neurodegeneration [137]. The amelioration of cognitive deficits is also partially induced by exercise’s favorable effect on methyl-CpG binding protein 2 (MeCP2) a reader of DNA methylation, and TETs as important mediators of hippocampus-dependent memory, such as LTP/LTD and excitatory synaptogenesis [136,138]. Certain epigenetic changes linked to accelerated aging may be prevented or delayed by exercise training [69]. The authors demonstrated that voluntary running wheel over eight weeks improved cognitive performance by downregulating Hdac3, and this process was linked to improved long-term memory and the recovery of contextual memory deficits in an Alzheimer’s disease (AD) mice model. A modulatory effect of activity on miRNAs in the hippocampal region was also found in this study. Other authors demonstrated that adult senescence-accelerated mice subjected to 4 weeks of regular training (running on a treadmill, 60 min/day, 5 days/week) display an increased level of hippocampal *Bdnf* and enhanced activity of Hat and Hdacs as a result of training [72].

Furthermore, oxidative stress is a major cause of neuronal cell death in neurodegenerative diseases. Antioxidant-related factors and enzymes, superoxide dismutase, catalase, and glutathione peroxidase play a role in protecting against oxidative damage. It was shown that DNA methylation is usually responsible for the reduced expression and function of antioxidant-related factors and enzymes such as nuclear factor erythroid 2-related factor 2 (Nrf2) and superoxide dismutase 2 (Sod2) in the course of neurodegeneration. Pretreatment with 4 weeks of swimming was able to induce the DNA binding activity of Nrf2 and expression of downstream antioxidant gene Sod2 in the hippocampal CA1 area in a streptozotocin (STZ)-induced sporadic AD rat model [139]. In the same brain structure, treadmill exercise (30 min/day, 5 days/week for 4 weeks) also effectively reduced oxidative damage in the STZ model, as evidenced by significantly lower peroxynitrite generation, lipid peroxidation, and oxidized DNA damage [140]. It seems that as a non-invasive strategy, physical activity can be incorporated as a component of therapeutic strategies in oxidative stress-based neurological disorders associated with epigenetic modifications.

There are many possible causes of neurodegeneration, but inflammation is a common factor in all of them. This process is initiated by microglia, which are the resident immune cells of the CNS, and can be triggered by different signals, and upon activation it may release pro-inflammatory molecules. Activation of NF-κB reduces *Tet* gene expression, leading to mild methylation aberrations. Additionally, the overproduction of nitric oxide (NO) during tissue inflammation upregulates Dnmt enzyme activity. Both of these conditions interact to promote aberrant methylation, which is an essential mechanism underlying the chronic inflammation in neurodegenerative disorders [141]. Also, Mecp2 epigenetically regulates microglia’s response to inflammatory stimuli. Mecp2 deficiency accelerates the shift of microglia into an active state, which then leads to microglia depletion. The impact of exercises on the signaling cascade is connected with the regulation of inflammatory response (mainly the TLR4, MyD88, NF-κB). Several studies examined whether the link between physical exercises and inflammation is mediated by epigenetic processes. According to Lovatel et al., [68] running for 20 min per day for two weeks improved aging-related cognitive impairment, reduced pro-inflammatory markers levels (IL-1b, TNF-a, phosphorylated NF-κB), and elevated histone H4 acetylation in hippocampi of 20-month-old Wistar rats. Such an exercise paradigm might reverse aging-related memory impairment through improvements in the cytokine profile. Another study found that treadmill exercise for 1 h per day, 5 days a week, activated the Trem2 pathway. This pathway inhibits microglial activation and neuroinflammation, leading to improved recognition memory in Alzheimer’s rat models induced by Aβ1-42 protein injections. Exercise improved AD-associated recognition memory via upregulating the Trem2 pathway, which enhances microglial phenotypic conversion and reduces neuroinflammation [142].

A growing body of evidence suggests that physical activity exhibits beneficial effects in alleviating the symptoms related to another neurodegenerative disorder—Parkinson’s disease (PD). During PD, progressive loss of dopaminergic cells in the substantia nigra and dopamine depletion in the striatum leads to malfunction in corticostriatal networks that are responsible for the control of movement and cognitive processes. Motor dysfunction frequently causes substantial and early difficulties in people suffering from PD and may serve as the main cause of disability [143]. Human studies demonstrated that aerobic exercise also improved cognitive disabilities, upregulated functional connection in the right frontoparietal network, proportional to fitness progress, and reduced total brain atrophy [144]. A randomized clinical trial indicated that the aquatic physical training program for 4 weeks (twice a week, 60 min each session) with different intensities induced functional improvement and Bdnf level enhancement at least partially by histone H4 hyperacetylation in the blood. This study emphasizes the importance of an active lifestyle for a constantly growing population with PD and the impact of peripheral Bdnf level improvement and histone acetylation on CNS modification [145]. Summing up, it seems that expanding knowledge about the role of epigenetic mechanisms in the aging process will lead to more effective therapeutic improvements (including physical activity programs for the elderly) in the prevention and treatment of age-related disorders.

## 4. Training Protocol as an Important Factor in Exercise-Induced Effects on the Brain

Currently, determining the most effective training protocol—encompassing exercise duration, intensity, and rest intervals—remains a crucial challenge. Achieving this requires large, homogenous participant groups to ensure reliable results. It was established that aerobic, anaerobic, and resistance training each yield different outcomes. However, the variability in training protocols and participant origin, sex, age, or health status across studies often make their results incomparable or unsuitable for meta-analyses and drawing informative conclusions. For example, daily exercise has been shown to increase histone H4 acetylation in the prefrontal cortices of 21-month-old rats without affecting younger rats. On the other hand, a single exercise session elevated Dnmt3b levels in the aged cortices of these animals [70]. The study indicates also that exercise-induced epigenetic changes may appear in a protocol-dependent manner. Another study on mice has demonstrated that endurance and resistance training activate distinct gene pathways. As a result of endurance exercise, mainly pathways related to neuroplasticity are activated, while on the other hand, an interferon response pathway in resistance exercise. Nevertheless, both are associated with enhanced learning and memory functions [67]. Therefore, scientists are currently confronted with a significant challenge: the selection of the most effective exercise protocol that is customized to the healthy person/patient’s age and capabilities, while simultaneously achieving the best potential health outcomes. In the context of research on epigenetic modifications of the CNS, animal models remain indispensable and their importance cannot be overstated.

## 5. Conclusions

Summarizing, it is well established that regular physical activity or exercise is beneficial to health and may diminish the risk for several diseases such as obesity, type 2 diabetes, cancer, and cardiovascular and neurological disease. Nevertheless, it also triggers numerous changes associated with greater brain function, i.e., cognitive processes, memory, and antidepressant properties. So far, the research on potential processes behind the impacts of physical activity on brain health mainly focuses on hormones, neurotrophins, and neurotransmitters, as well as intra- and extracellular pathways. Recently, they have taken into account altered gene expression profiles by epigenetic modifications, DNA methylation, alterations in histones, and epigenetic changes involving microRNAs as well as key exercise-responsive genes, which were for a long time studied mainly in peripheral tissues, especially skeletal muscles. Recent studies on the brain indicate that the impacts of exercise are more persistent than previously assumed and may affect future generations. Exercise’s effect on epigenetic regulation of gene expression appears to be critical in developing an “epigenetic memory” that influences long-term brain function and behavior. So far, most of the available data concern running training, so in the era of access to various forms of training, expanding this type of research is required. Additionally, the duration of beneficial effects is still not fully established; robust correlation studies between training frequency, morpho-functional changes, and impacts on brain function over time are essential. Because the influence of exercise varies based on age and stage of life, it is important to consider when their benefits are most advantageous. Moreover, since the regulation of the epigenetic mechanism may prevent the development or delay the progression of neurological and mental diseases, epigenetic therapy of CNS diseases seems to be a promising field in pharmacotherapy. However, since epigenetic neuropharmacology is still a relatively new field, the studies on drug properties that interfere with epigenetic machinery (such as antiepileptic, tricyclic, and selective serotonin reuptake inhibitor antidepressants, and drugs used in neurodegenerative disorders or addiction) are at an early stage (reviewed in [146]). It also appears that the comparison of the effects of treatments that regulate the epigenome and various forms of physical activity may be beneficial in the context of prevention as well as (co)therapy. This is relevant because therapy with currently known epi-drugs faces several challenges (i.e., difficulties with permeability through BBB, compound activity, and complicated genome–epigenome interactions). However, designing personalized recommendations for physical activity is also demanding because the epigenetic arrangement of a great number of genes influences, in different ways, both an individual’s ability to exercise and the outcomes of that exercise. Nevertheless, therapy with currently known epi-drugs faces several challenges (i.e., difficulties with permeability through BBB, compound activity, and complicated genome–epigenome interactions). Therefore, noninvasive, non-pharmacological prevention or treatment for these diseases with the use of exercises as lone or complementary factors seems very promising.

## Figures and Tables

**Table 1:** Studies demonstrating the impact of physical exercise at different stages of life on epigenetic changes observed in the offspring’s brain.

Study	Time of Exercise Intervention	Model (Animals)	Exercise Protocol	Epigenetic Changes Observed in the Brain
preconception period				
Mega et al., 2018 [49]	preconception, paternal	Wistar rats, male	treadmill running; 20 min/day, 5 consecutive days/week, 8 weeks	decrease in global hippocampal DNA methylation level in rat offspring
Spindler et al., 2019 [50]	preconception, paternal	Wistar rats, male	treadmill running; 20 min/day, 5 consecutive days/week for a total period of 22 training days	decrease in global hippocampal DNA methylation level in rat offspring
Segabinazi et al., 2019 [51]	preconception, maternal	Wistar rats, male	treadmill running; 20 min/day, 5 consecutive days/week for a total period of 22 training days; exercise 30% VO_2_ max	decrease in global hippocampal DNA methylation level in rat offspring
Meireles et al., 2021 [52]	preconception, maternal	Wistar rats	resistance exercise; voluntary vertical ladder climbing, with lead weights fixed to an animal tail	decrease in global hippocampal DNA methylation level, increase in global hippocampal H4 acetylation in offspring
Luft et al., 2020 [53]	preconception, maternal	Balb/c mice, male	treadmill running; 10 m/min for 60 min, 5 days/week, 3 weeks	decrease in histone H3 acetylation (*p* = 0.06) in offspring
gestational period				
Wu et al., 2020 [54]	prenatal	Sprague Dawley rats, male	treadmill training; 60 min/day, 5 days/week, 3 weeks, 8 m/min for 5 min, followed by 10 m/min for 25 min and then 12 m/min for 30 min	amelioration of the reduction in p300 Hat expression and histones acetylation (ace-H3K14, ace-H3K27) in the hippocampus of offspring
Meireles et al., 2021 [52]	prenatal	Wistar rats, male	resistance exercise—climbing voluntarily a vertical ladder, with lead weights fixed to an animal tail	decrease in the level of Hdac2 in the hippocampus of offspring
early life period				
Collins et al., 2009 [55]	young, male	Sprague-Dawley rats, male	voluntary wheel running for 4 weeks	increase in histone H3 phospho-acetylation
Intlekofer et al., 2013 [56]	6-weeks-old, male	C57Bl/6J mice	voluntary wheel running for 3 weeks	hyperacetylation of H4K8Ac of *Bdnf* I and IV promoters leading to increased *Bdnf* transcript I and IV levels
Abel et al., 2013 [57]	46-day-old, male	C57BL/6J male mice	voluntary wheel running for 7 days	increase in global histone H3 acetylation in the hippocampus and cerebellum, correlated with upregulation of Bdnf in the hippocampus; decrease in the expression pattern of *Dnmts* and *Hdacs* in both areas after exercise
Raus et al., 2023 [58]	PND21-PND41	Emx1-NuTRAP mice, male	voluntary wheel running for 20 days	the occupancy of H4K8ac and H3K27me3 at regulatory regions of genes that are involved in the consolidation of hippocampal memory was altered by ELE
adult life				
Gomez-Pinilla et al., 2011 [43]	3-month-old	Sprague-Dawley rats, male	voluntary wheel running	DNA demethylation at the *Bdnf* promoter IV; increased Mecp2 protein level and histone H3 acetylation, and reduction of *Hdac5* transcript and protein level in the hippocampus
Elsner et al., 2011 [44]	2–3 months-old	Wistar rats, male	treadmill running, single session: 20-min or chronic protocol: 20-min running session each day for 2 weeks	decrease in Hdac activity, increase in Hat activity, and Hat/Hdac balance in male rat hippocampi induced by a single training session
Elsner et al., 2013 [59]	3-months-old	Wistar rats, male	two exercise protocols: single session of treadmill exercise: 20 min, and chronic treadmill protocol: 20 min running session each day for 2 weeks	decrease in both Dnmt3b and Dnmt1 levels in male rat hippocampi induced by the single exercise session; both exercise protocols reduced H3K9 methylation level in this structure
Sleiman et al., 2016 [60]	-	C57BL/6 mice, male	voluntary wheel running for 30 days	reduced levels of Hdac2 and Hdac3 in the hippocampus
Bao et al., 2014 [45]	4–4.5-month-old	C57BL/6J mice subjected to traumatic brain injury, male	voluntary wheel running for 2 weeks	altered miRNA expression in the hippocampus: miR-21 and miR-34a
Patki et al., 2014 [61]	adult	Sprague-Dawley rats, Social defeat stress model, male	treadmill running, 2 weeks, 30 min/day	normalization of the alterations in male rat hippocampi caused by stress on histone H3 acetylation, Hdac5, and Mecp2
Ieraci et al., 2015 [62]	9-weeks-old	Male C57BL/6J mice, male	voluntary wheel running for 4 weeks	increase in histone H3 acetylation at all *Bdnf* promoters except for *Bdnf*-8
Kashimoto et al., 2016 [63]	PND53-PND78	Wistar rats, male	swimming exercise (5 days a week and 60 min/day) followed by repeated restraint stress during 75–79 PND	increase in global DNA methylation in the hippocampus, cortex, and hypothalamus; decreased expression of the *Dnmt1* gene in the hippocampus and hypothalamus
Zhong et al., 2016 [64]	3-months-old	C57BL/6 mice, male	swimming for 4 weeks, 4 sessions of 5 min/day, 3 months after isoflurane exposure	enhanced hippocampal H3K9, H3K14, H4K5, H4K8, and H4K12 acetylation levels
Zhao et al., 2019 [65]	2-month-old	Wistar rats, male	6-week training program, 10 sessions of swimming training/day, 6 min each with a 4-min rest between sessions	34 miRNAs differentially expressed in response to HIST; 16 were up-regulated and 18 were down-regulated; upregulation of all miR-200 family members
Vetreno et al., 2020 [66]	PND56-PND95	Wistar rats, male	voluntary wheel running for 40 days	restoration of the increase in H3K9me2 and DNA methylation at the promoter regions of Chat and Trka caused by adolescent intermittent ethanol
Urdinguio et al., 2021 [67]	8-weeks-old	C57BL/6N mice, male	treadmill running; 60 min /day, 5 days/week, 4 weeks; resistance training: climbing a ladder with a weight load	decrease in the overall DNA methylation status in the hippocampus
aging and neurodegeneration				
Elsner et al., 2013 [59]	20 months old	Wistar rats, male	two exercise protocols: single session of treadmill exercise (20 min) and chronic treadmill protocol (20 min running session each day for 2 weeks)	reverse of the changes in H3-K9 methylation levels induced by aging as a result of the single session of training
Lovatel et al., 2013 [68]	20-months-old	Wistar rats, male	treadmill exercises—rats ran at 4.2 m/min for the first 4 min, 9.5 m/min for 12 min, and 4.2 m/min for the last 4 min	amelioration of the age-related decrease in histone H4 acetylation level in the hippocampus
Cosin-Thomas et al., 2014 [69]	8-months-old	senescence-accelerated SAMP8 mice (AD model), females	voluntary running wheel for 8 weeks	modulation of *Hdac3* and *Hdac5* genes expression, reduction of the global histone H3 acetylation levels in SAMP8 and normalization in response to exercise; exercise-induced alteration in the expression of seven miRNAs (upregulated: miR-28a-5p, miR-98a-5p, miR-148b-3p, miR-7a-5p and miR-15b-5p; downregulated: miR-105, and miR-133b-3p) in the hippocampus
Cechinel et al., 2016 [70]	21-months-old	Wistar rats, male	single session (20 min) or daily moderate 20 min/day for 14 days	increase in histone H4 acetylation levels in the prefrontal cortex after the daily exercise protocol; increase in the level of Dnmt3b in aged cortices of animals submitted to a single session of exercise
Jessop et al., 2018 [71]	18-months-old	C57BL/J6 mice, male	voluntary wheel running	normalization of aging-related decreased levels of translocation methylcytosine dioxygenases (*Tet1* and *Tet2*)
Maejima et al., 2018 [72]	13-months-old	13-month-old SAM resistant 1 (SAMR1) and SAM prone 1 (SAMP1) lines	running on a treadmill; 60 min/day, 5 days/week, 4 weeks	enhanced activity of Hat and Hdacs in the hippocampus
Ionescu-Tucker et al., 2021 [73]	18-months-old	C57BL/6J mice	voluntary wheel running, 6 weeks	decrease in H3K9me3 at *Bdnf* promoter VI
